# Advancing Research on Proprotein Convertase Subtilisin/Kexin Type 9 Inhibitors: A Scientometric Analysis

**DOI:** 10.21315/mjms2024.31.4.2

**Published:** 2024-08-27

**Authors:** Abdul Matin, Gul-e-Saba Chaudhry, Mohamad Nor Azra, Mohamad Gazali, Yik Sung Yeong, Tengku Sifzizul Tengku Muhammad

**Affiliations:** 1 Institute of Climate Adaptation and Marine Biotechnology, Universiti Malaysia Terengganu, Terengganu, Malaysia; 2 Department of Food Processing and Engineering, Faculty of Food Science and Technology, Chattogram Veterinary and Animal Sciences University, Chattogram, Bangladesh; 3 Department of Marine Science, Faculty of Fisheries and Marine Science, University of Teuku Umar, West Aceh, Indonesia

**Keywords:** atherosclerosis, bempedoic acid, PCSK9, PCSK9 inhibitors, lipid-lowering therapy

## Abstract

Atherosclerosis is characterised by the accumulation of fatty deposits and plaque as a result of a continuously high level of low-density lipoprotein cholesterol (LDL-C) in the blood. The primary objective of this research is to assess the current status of knowledge, research endeavours and developmental trajectories about proprotein convertase subtilisin/kexin type 9 (PCSK9) inhibitors in correlation with atherosclerosis treatment. Additionally, this study aims to compile bibliometric and scientometric investigations within this domain through rigorous scientometric analysis. Analysing the bibliometric landscape and global research trends associated with PCSK9 inhibitors can contribute valuable insights into comprehending atherosclerosis. This is exemplified by examining publications within the Web of Science Core Collection (WOSCC) database from 2008 to 2022. Citespace was used for frequency, co-occurrence, co-citation, grouping and burst analysis, and Microsoft Excel was used to manage descriptive datasets. Eight hundred eighty-five publications available from WOSCC database between the years 2008 and 2022 were extracted and examined. Over the period, 3,138 collaborating institutions from 87 countries, a staggering 7,750 writers involved and 325 distinct journals published about PCSK9 inhibitors studies. Among authors, Sabatine et al. and the journal *The New England Journal of Medicine* has had the most significant impact. Lipid-lowering therapy and bempedoic acid are the most prominent topical clusters associated with PCSK9 inhibitors, and the most often used keywords are efficacy, safety and PCSK9 inhibitors. We believe this is the first comprehensive analysis of PCSK9 inhibitors research and publications conducted using Scientometric. These results demonstrate the nascence of PCSK9 inhibitors research. They may encourage a wide range of stakeholders, particularly early career researchers from various disciplines, to work together in the future.

## Introduction

Worldwide, cardiovascular disease is among the leading killers. For cardiovascular patients, coronary heart disease (CHD) has the highest yearly mortality rate (1). Atherosclerosis can be caused by a number of well-known CHD risk factors, such as high blood cholesterol and triglyceride levels (2–4). The aim of the research is to evaluate the present state of knowledge, research and development trends in proprotein convertase subtilisin/kexin type 9 (PCSK9) inhibitors in conjunction with the treatment of atherosclerosis and collect bibliometric and scientometric studies of the domain through scientometric analysis. The bibliometric landscape and global research trends related to PCSK9 inhibitors might be helpful in understanding atherosclerosis, as evidenced by publications in the Web of Science Core Collection (WOSCC) database between 2008 and 2022. PCSK9 is a serine protease that is produced in the liver. It can either be maintained in the cytoplasm or secreted into the blood, which is important for controlling the life cycle and the quantity of low-density lipoprotein receptors (LDL-Rs) (5). Consequently, lowering plasma levels of low-density lipoprotein cholesterol (LDL-C), which is still the foundation in atherosclerosis treatment, by the inhibition of PCSK9 activity presents a promising therapeutic target (5–11). The chylomicrons are formed from dietary fat and apolipoproteins B (ApoB) under normal physiological conditions; these are then lipolysed to hydrolyse triglycerides and deposited in adipose tissue for later use as fuel (4). By combining with apolipoprotein E (ApoE) coupled to LDLR, chylomicron remnants gain access to the liver (4). In addition, liver-secreted very low-density lipoproteins (VLDL) were converted to intermediate-density lipoproteins (IDL) (12). IDL can attach to ApoE and form a complex with LDLR or it can undergo further hydrolysis and conversion into LDL-C (4). In a typical circumstance, when LDL-R binds to LDL-C, the complex is endocytosed into liver cells. Following the release of LDL-C, LDL-R returns to the cell surface. Once more, the receptors bind to more circulating LDL-C particles (10). In 2003, Seidah et al. (13) identified the ninth member of the proprotein convertase family, PCSK9. The intervention of PCSK9 in regulating cholesterol metabolism was revealed the same year, with the discovery of two gain-of-function (GOF) mutations in PCSK9 in two French families with a medical assessment of antidiuretic hormone and no detectable mutations in LDLR or apoB100 genes (14). After apoB100 and LDL-R, it is the third gene linked to autosomal dominant familial hypercholesterolemia (15). The secreted form of PCSK9, on the other hand, has the ability to attach to LDL-R and direct it toward lysosomal breakdown, which stops the receptor from being recycled back to the cell surface. Moreover, the PCSK9 enzyme helps boost the breakdown of LDL-R inside of cells by way of an intracellular mechanism that is only partly understood (4). As a result, PCSK9 lessens the quantity of LDL-R on hepatocytes’ cell surfaces. Therefore, elevated levels of PCSK9 in the blood can lead to higher levels of LDL-C in the blood, and as plasma LDL-C is the most important factor in the development and progression of atherosclerosis, high levels of PCSK9 can contribute to the disease (5, 16, 17). The scientometric investigation, a visually represented statistical way of analysing available literature, is one of the utmost commonly used approaches for finding up-to-date trends and research gaps in a database (18–20). But, as yet, no scientometrics study of PCSK9 inhibitors research has been conducted. One way to evaluate the present state of knowledge, research, and development trends in PCSK9 inhibitors and collect bibliometric and scientometric studies of the domain is through scientometric analysis. Consequently, the following questions will be addressed in this study to accomplish this purpose. What are the general publication drifts in terms of output? What are the key sources of information in these fields? What are the predominant topics/clusters and how have they changed over time? What influential articles and keywords are relevant to these fields? Researchers, students and the academic community can use this study’s findings to understand better the future of PCSK9 inhibitors research and its connection to cardiovascular disease, as well as to detect emerging patterns and present obstacles in this field. Several stakeholders would benefit from being apprised of recent advances in PCSK9 inhibitors research. For instance, the submission rate and the journal’s eligibility for the PCSK9 inhibitors research study are both affected by the number of journals in which the authors have previously published. As a result, the time spent looking for appropriate journals will be cut down. The publication of a list of highly regarded journals that welcome well-written papers in this area will help pique the interest of many more scholars.

## Methods

**Figure 1:**
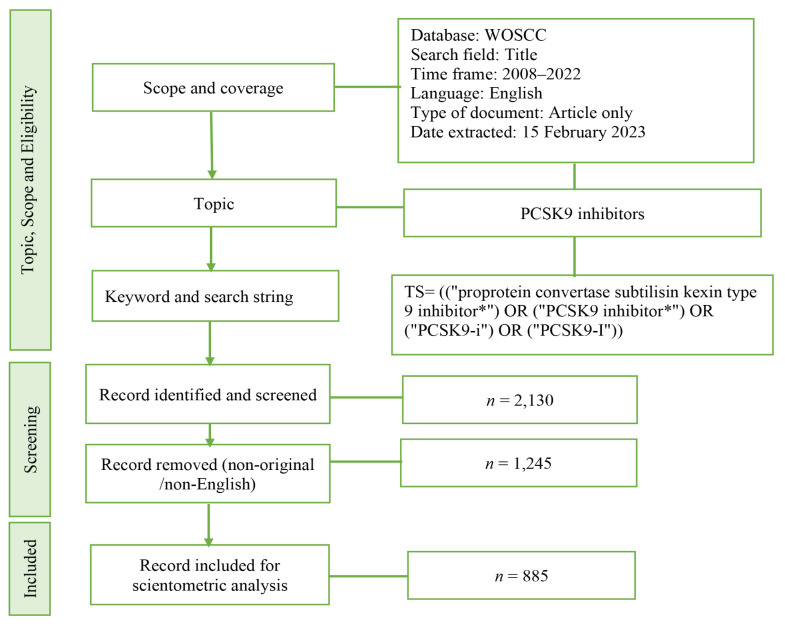
The present study’s flowchart or methodological framework on PCSK9 inhibitors

The research outline used in this study is revealed in Figure 1. The figure represents the methodological framework on the PCSK9 inhibitors. Briefly, the Thomson Reuters IS WOSCC database was used to find literature online. PCSK9 inhibitors were the main topic for the article search as mentioned earlier. The data was extracted on 15 February 2023 and only the article which was written in English was included. The first paper appeared on 2008. A total of 2,130 record was found on this topic where 1,245 was removed. Finally, a total of 885 original articles written in English were included in the scientometric analysis.

### Data Origin

The Thomson Reuters IS WOSCC database was used to find literature online. The topic search (TS) field, which comprises article titles, abstracts, keywords and ‘keywords plus’, obliged as the source for the WOSCC database searches (automatically created terms drawn from the titles of mentioned articles). Current studies claim that WOSCC is one of the top scientific databases, covering approximately 34,000 articles and containing more than 1.8 billion referenced references. It was also covered by numerous academic disciplines and included in indexed peer-reviewed publications with high-quality journals (18, 19, 21).

### Article Search

Based on keywords recommended by the WOSCC and online literature from previous studies, related keywords for PCSK9 inhibitors were identified. The search occurred on 15 February 2023 (https://www.webofscience.com/wos/woscc/summary/4c53293e-2309-44f4-ad94-2503498569df-708fdb70/relevance/1). The search term is shown as:

TS = ((“proprotein convertase subtilisin kexin type 9 inhibitor*”) OR (“PCSK9 inhibitor*”) OR (“PCSK9-i”) OR (“PCSK9-I”))

### Eligibility Principles

#### Inclusion Principles

Only English-language publications that were published in peer-reviewed journals were taken into consideration for inclusion in our future analyses after they were retrieved via WOSCC database searches.

#### Exclusion Principles

We did not include an article if it was not considered novel research, issued in a publication that did not undertake peer review, or written in a language other than English. These studies may have encompassed book reviews, proceedings articles, editorials, reviews, abstracts, letters, or bulletin.

### Data Investigation

Excel was used to show vivid analyses for the quantity of publications, published journals, authors, universities/institutes and countries/provinces where the authors were associated and resided at the period the articles were issued to examine publishing output patterns.

#### Scientometric Analysis

CiteSpace, v. 6.1.R6 (advanced) for 64-bit Windows was utilised for imagining and knowledge diagram analysis since it agrees for building different bibliometric networks and using multiple analysis techniques (22, 23).

The threshold was set at ‘g-index (scaling factor, k =25)’ for each slice, allowing the assortment of the most cited matters to construct a network grounded on input value and numerous node kinds. ‘Time slicing’ was set to 2008–2022 and ‘years per slice’ was set to one year. The ‘pruning’ parameter was used to trim the produced network. All term sources in WOSCC were picked for text processing, including title, abstract, author keywords and keywords plus (21).

#### Co-Citation Analysis

The state of systematic progress and shifts in scientific assembly can be ascertained by co-citation analysis. The co-citation analysis produces a science map with nodes, connections and density values to depict the main form of selected variables (for this study: i) author, ii) journal publication, iii) countries, iv) institutions, v) research topics and vi) articles) to obtain the co-citing variable cluster, where a co-citation instance occurs when two sources are cited together in the same paper. A red ring around a node denotes an exponential rise in citations for a particular article. Generally, Citespace constructs a co-citation network based on the citation links between publications. Each node represents a publication and the edges represent co-citation relationships. Citespace provides various analysis and visualisation options to explore co-citation patterns, such as generating visual maps to reveal clusters of co-cited publications, identifying influential articles and detecting emerging research topics (18, 19, 21, 23, 24).

#### Document Cluster Analysis

Multidimensional clustering was applied to the obtained documents to separate similar research pieces into distinct categories. The log-likelihood ratio (LLR) was utilised to automatically extract the cluster label since it may deliver the best results regarding uniqueness and coverage. The ‘timeline view’ and ‘cluster view’ visualisation options in Document Cluster Analysis were employed to see how the network was structured. Whereas the ‘timeline view’ presented a horizontal span of time from left to right, the ‘cluster view’ showed a landscape-oriented spatial network of colour-coded and automatically annotated representations. Citespace constructs a document co-citation network based on the co-occurrence of citations between documents. Briefly, Citespace applies a clustering algorithm to group related documents based on their citation patterns. Different algorithms, such as VOS clustering, can be used to identify document clusters. Also, Citespace visualises the document clusters using various visualisation techniques, such as overlaying the clusters on a co-citation network or representing them as distinct groups. These visualisations help identify thematic clusters within a research field (18, 19, 21, 23, 24). Each term’s specifics are defined on the official CiteSpace homepage (https://citespace.podia.com/glossary). The average silhouette metric, modularity Q index and centrality metric were utilised to evaluate the superiority and homogeneousness of the article cluster studies in addition to the found clusters (24, 25). The modularity Q index goes from 0 to 1, with bigger values denoting greater dependability. The average silhouette metric ranges from −1 to 1, with values larger than 0 suggesting better uniformity. Centrality is a metric of the impact that illustrates how close publications or journals are to one another, with publications with a high centrality having a greater impact on the network since they attach more magazines or journals. Therefore, more information and pathways flow through them.

#### Burstness Analysis

To select influential articles and top keywords, citation burstness and sigma, both temporal measures, were applied. A burst detection is described as ‘an abrupt elevation in the frequencies (of citations) over a specified time frame,’ as represented by a red circle around the node. CiteSpace applies a burst detection algorithm to identify periods of intense citation activity for specific publications. The algorithm looks for clusters of citations that occur within a shorter time span compared to neighbouring citations. Then, CiteSpace calculates the strength of each burst based on the number of citations and their distribution over time. The strength indicates the intensity of the citation burst (18, 19, 21, 24, 25).

#### Knowledge Map Identification

In this part, the parameters for knowledge map identification techniques will be presented. The node is a metric used to fix the incidence with which a variable has been referenced, where a big node specifies a high figure of citations and the line connecting the nodes shows that these nodes exist in the same articles. CiteSpace constructs a co-citation or citation network using the bibliographic data. Then, CiteSpace analyses the network to identify clusters or groups of related publications. It provides visualisations, such as knowledge maps, to represent the intellectual structures and relationships among different research topics. Centrality refers towards the influence that shows the degree of measurement to which the same variables are close to each other, with high centrality having a greater influence on the network because they connect more variables and thus more information and paths pass through them. ‘Degree’ refers to the number of links between the different variables in the information map. A higher ‘degree’ value indicates greater communication and collaboration between the various variables (e.g. institutions, countries). The red rings encircling the node revealed its bursting nature. It shows where the items begin to ‘burst’ and the strength of the ‘burst’ force. The purple rings represented a node’s centrality; a node with a high centrality specified a tactical position and the capacity to connect to other nodes in the selected networks (19).

## Results

### Descriptive Data

#### Progression of Publications

**Figure 2:**
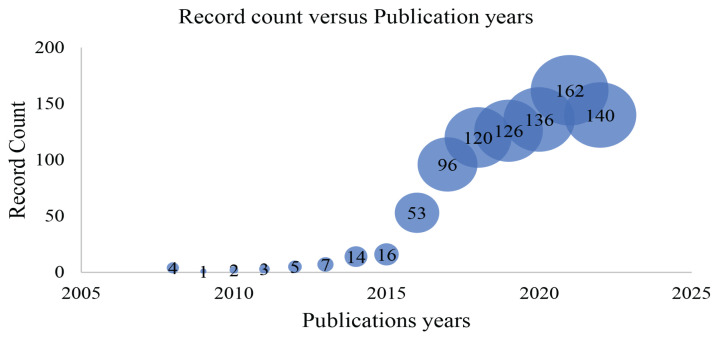
Publication trends of PCSK9 inhibitors search between 2008 and 2022, with 2016–2022, the number of documents shot up quickly

The research was restricted to scientific documents published between January 2008 and December 2022 (Figure 2). A total of 885 articles were composed during this period. After 2018, the number of publications gradually augmented, with 684 articles published in the last 5 years between 2018 and 2022.

#### Productive Authors

Thirty-one authors have published over 15 publications on PCSK9 inhibitors since 2008 (Table 1). Two thousand six hundred seventy-five authors (35.43%) contributed more than one article out of 7,550. Sabatine MS had the most publications, followed by Giugliano RP and Mach F.

#### Topmost Institutions

There were 3,138 institutions involved in this research all over the world. The topmost ten institutions in terms of whole publications are shown in Table 1—the top three affiliations from the United States of America. Harvard University was the most productive publisher, with 102 publications, followed by the Brigham and Women’s Hospital (84 publications), Amgen (78 publications) and the fourth is Sanofi Aventis in French (75 publications). The top 10 institutions issued 687 (77.63%) articles on average.

#### Industrious Journals

The topmost 10 journals by the number of publications are shown in Table 1. We found eligible articles in 325 journals in total. *Journal of Clinical Lipidology* had the most publications (61 publications), followed by *Atherosclerosis* (36 publications), *Journal of the American College of Cardiology* (23 publications) and *Journal of the American Heart Association* (23 periodicals). Between 2008 and 2022, the top 10 journals published 241 (27.23%) articles.

#### Country Distribution

**Figure 3:**
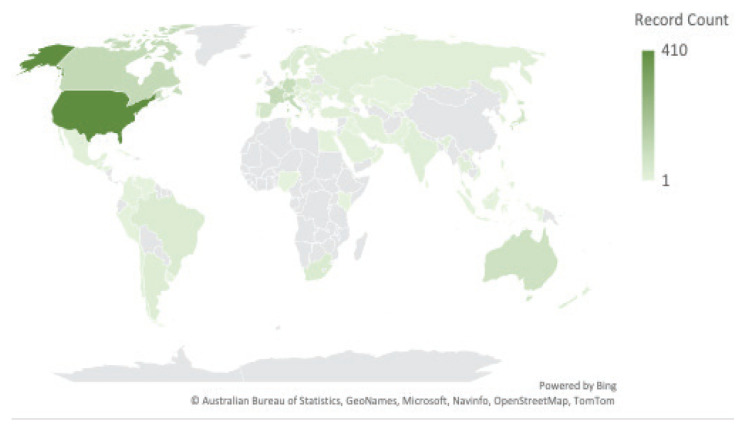
Regarding regional and state distribution of PCSK9 inhibitors researches worldwide, darker shades of green indicate more overall publications, while lighter ones indicate fewer publications

**Figure 4:**
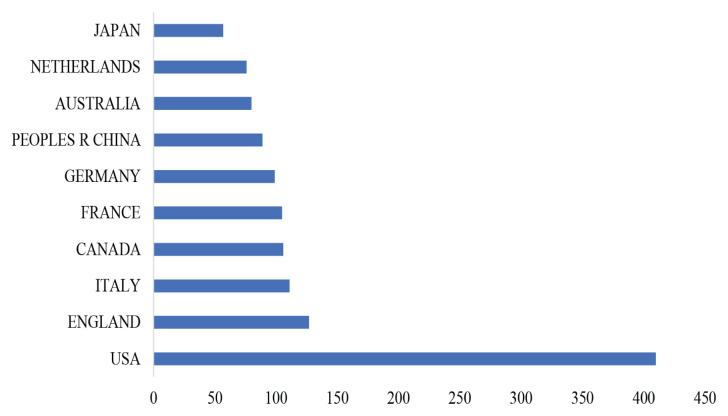
Number of publications issued between 2008 and 2022 from the topmost 10 countries

**Figure 5:**
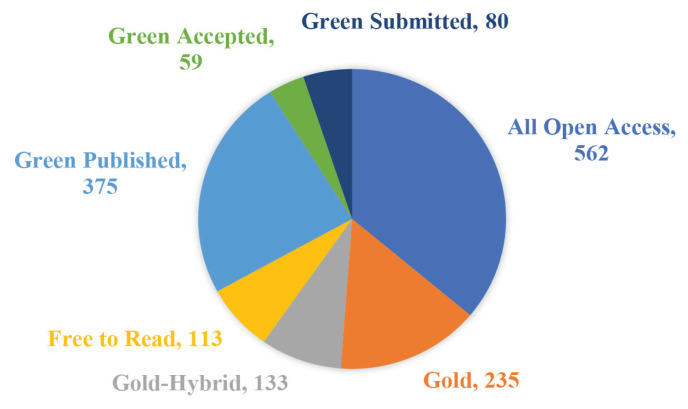
Level of open access to the PCSK9 inhibitors-related literature written between 2008 and 2022, based on the WOSCC database

Figure 3 shows the regional and state distribution of PCSK9 inhibitors research worldwide. The topmost 10 countries and regions with the utmost publications are shown in Figure 4. Eighty-seven countries had relevant publications. The United States of America had the most publications (410), followed by England (127) and Italy (111 publications). According to the WOSCC database, all papers were open-access (Figure 5). Figure 5 shows that there were 562 articles that have open access. Around 235 articles have a Creative Commons (CC) license and are referred to as Gold whereas Hybrid Gold also has a CC license different from the Gold journals, especially for newly published articles. A publisher may as a promotion, grant free access to an article for a limited time which is referred to as ‘free to dead’. Green Published articles are final published versions of articles hosted on an institutional or subject-based repository whereas Green Accepted are the accepted manuscripts hosted on a repository. Green Submitted are the original manuscripts submitted for publication but that has not been through a peer review process.

### Scientometric Study

#### Co-Citation Analysis

Using nodes, linkages and density values, co-citation analysis creates a science map that shows the primary assembly of a variable’s development, position and changes through time. The findings of the co-citation analysis are shown below for the article document, author, journal, nation or region, institution and research topics.

#### Author Co-Citation Analysis

**Figure 6:**
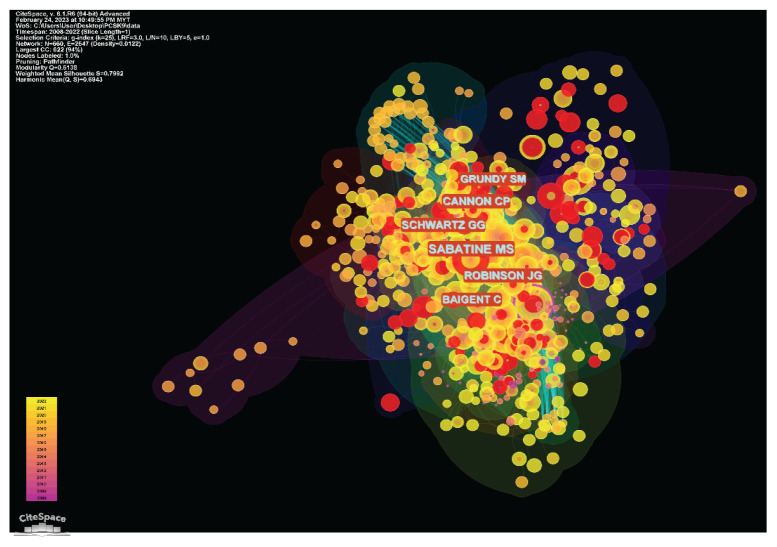
An author co-citation analysis

The author’s co-citation network had 660 nodes and 2,647 connections. The density of the co-citation network was 0.0122. The modularity Q index and the average silhouette metric for the author co-citation network in the existing study were 0.6138 and 0.7992, respectively, suggesting that the author network had high degrees of homogeneity and connectedness. The topmost 10 cited authors with the utmost frequency in these arenas are listed in Table 9. The frequency of Sabatine et al. in the foremost place is 483, the first time to appear in a supportive relationship in 2015, followed by Robinson et al. with a frequency of 298 and its first presence being 2015, and Schwartz et al. (30) with a frequency of 292 and first appearance being 2013 is on third place. Their collaboration history can be seen in Figure 6. Each node denotes an author, and the larger the node, the more frequently that author has been quoted. The red rings encircling the node revealed its bursting nature. It indicates where the items begin to ‘burst’ and the strength of the ‘burst’ force. The purple rings represented a node’s centrality; a node with a high centrality specified a tactical position and the capacity to connect to other nodes in the selected networks. Other scholars’ cooperation frequency is all above 150 and the time they began collaborating with other scholars is close to the same years. That suggests that the researchers working on the PCSK9 inhibitors study are more aggregated and have strong academic ties.

#### Countries/Region Co-Citation Analysis

There are 87 nodes, 778 connections and a density of 0.208 in the co-citation network for countries/regions. Figure 9 reveals a network of co-citations grounded on the lead writers’ countries/regions. Each node characterises a country and the larger the node, the more often that country has been mentioned. The red rings around the node suggested that it was bursting. It shows where things start to ‘burst’ and how robust the ‘burst’ force is. The purple rings showed the centrality of a node; a node with a high centrality specified a tactical position and the ability to connect to other nodes in the chosen networks. This result is also consistent with the findings in Figure 4.

#### Journal Co-Citation Analysis

**Figure 7:**
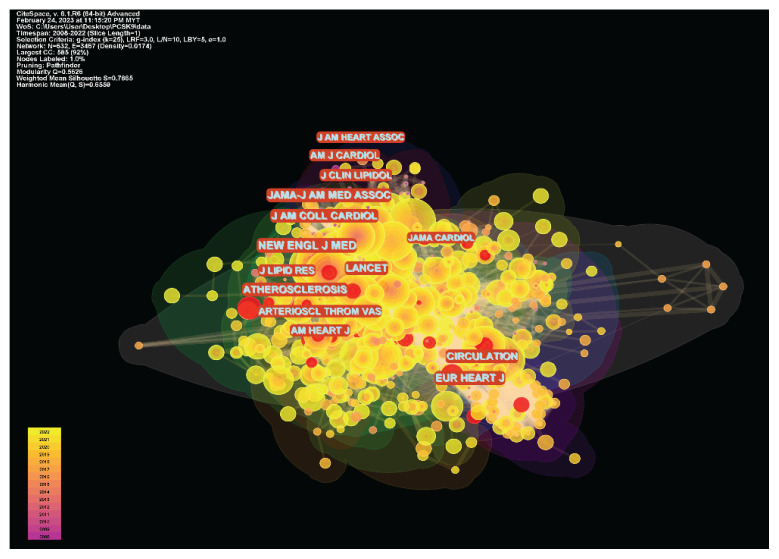
The knowledge maps for journal co-citation analysis

The journal co-citation network had 632 nodes and 3,467 connections. The density of the co-citation network was 0.0174. The knowledge maps for journal co-citation analysis are shown in Figure 7. The modularity Q index and the average silhouette metric for the author co-citation network in the present study were 0.5626 and 0.7865, respectively, suggesting that the journal network had high degrees of homogeneity and connectedness. The top 10 cited journal with the most frequency in these fields is listed in Table 2. With a frequency of 711, *The New England Journal of Medicine* was the most influential. *Circulation* (frequency: 618) and *Journal of the American College of Cardiology* (frequency: 613) were the second and third most influential journals.

**Figure 8:**
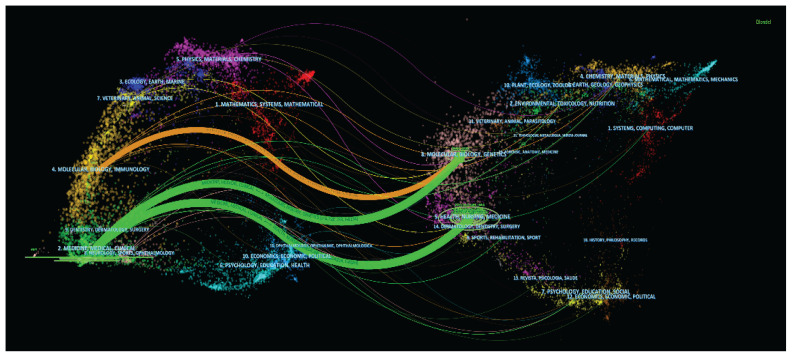
An overlay of the cited journal in PCSK9 inhibitors research

**Figure 9:**
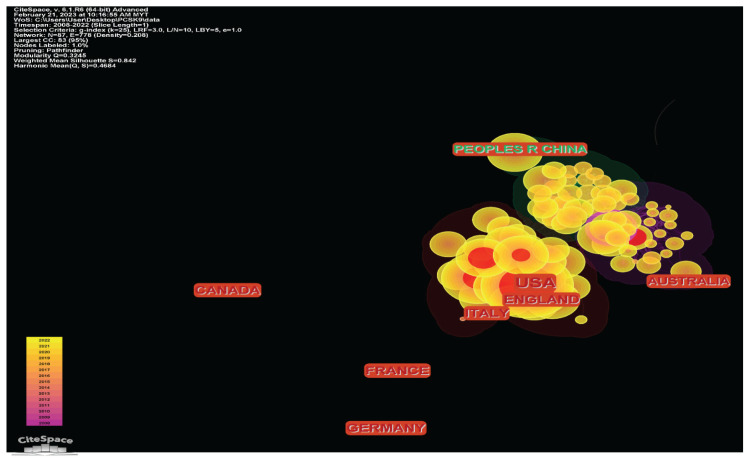
Network of country/region co-citation analysis

The interdisciplinary confluence of disciplines is necessary since research topics are becoming more complicated. Figure 8 displays an overlay of the mentioned journal. Each dot in the diagram stands for a journal and the sum of each colour symbolises the related topic. The graph’s left side displays the topic distribution of the cited article, while the right side displays the topic distribution of the referenced article. The base map contains information on Mathematics, Medicine, Ecology, Molecular, Physics, Psychology, Animal Science and so on. The placement of the ellipse also depicts the diversity of the study’s participating fields. The vertical axis of the ellipse explains the number of articles, while the horizontal axis explains the number of authors. The current view depicts the z-score function and the connecting lines’ thickness indicates the relationships’ relevance. The image includes two distinctive wavy lines that flow from the left to the right of the oval in the illustration. The green wavy line illustrates the interdisciplinary cross-reference between Medicine, Medical and Clinical, not only with Health, Nursing and Medicine but also Molecular, Biology and Genetics. At the same time, the brown wavy lines connect, citing articles and cited articles representing Molecular, Biology and Immunology in this study.

#### Institution Analysis

**Figure 10:**
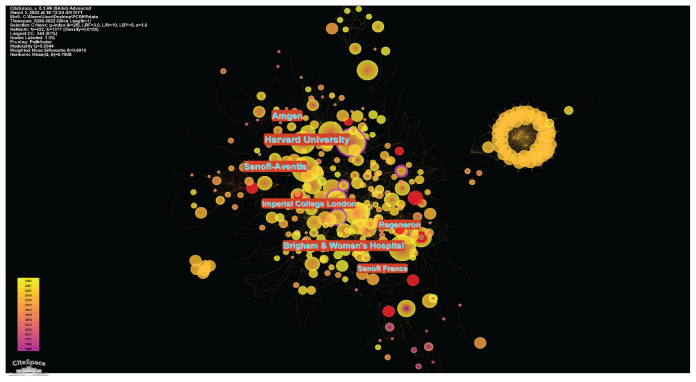
Network of institutions co-citation analysis

The institution co-citation network had 442 nodes and 1,377 connections. The density of the co-citation network was 0.0155. The modularity Q index and the average silhouette metric for the author co-citation network in the present study were 0.6944 and 0.8916, respectively, suggesting that the institution network had high degrees of homogeneity and connectedness. Figure 10 depicts a network map of institutional collaboration, with each node in lieu of an institution and the lines between them representing citation-based collaboration. The size of the node indicates how many documents the organisation has produced. The organisation per larger node distribution implies more papers. The largest of them all is the node for Harvard University. Also, the linkages show how the organisations cooperate. The cooperation between the organisations gets closer, the more ties there seem to be. The red rings encircling the node revealed its bursting nature. It indicates where the institution begins to ‘burst’ and the strength of the ‘burst’ force. The purple rings represented a node’s centrality; an institution with a high centrality indicated a strategic position and the capacity to connect to other institutions in the selected networks. The top 10 institutions with the most frequency in these grounds are enumerated in Table 4. Furthermore, among the 10 revealed institutions in Table 3, seven were from the United States, two were French and one was from England.

#### Web of Science Study Area Co-Citation Analysis

**Figure 11:**
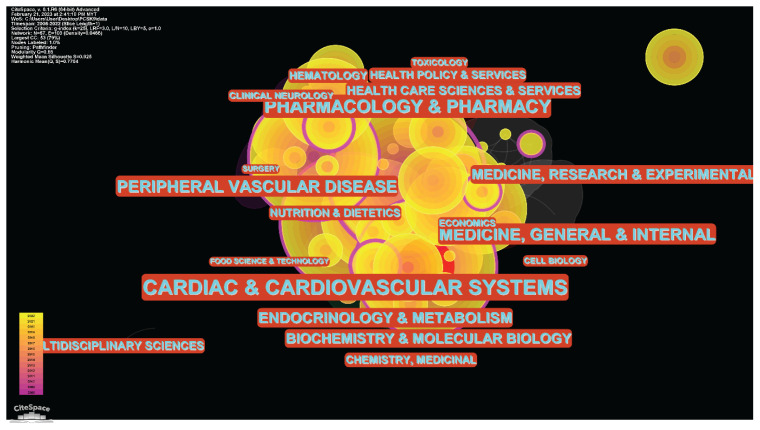
Network of study area co-citation analysis

The Web of Science study areas co-citation network had 67 nodes and 103 connections. The density of the co-citation network was 0.0466. Figure 11 illustrates a network map of co-citation between study areas, with each node on behalf of a study area and the lines representing citation-based cooperation. The node size indicates how many documents the research area has covered. The research area per larger node encloses more papers. The largest of them is the Cardiac and Cardiovascular Systems node. Also, the linkages show how the research areas cooperate. As connections strengthen, researchers in different fields will work together even more closely. The red rings encircling the node revealed its bursting nature. It indicates where the institution begins to ‘burst’ and the strength of the ‘burst’ force. Centrality was represented by the size of the purple rings surrounding each node; a research area with a high centrality was in a prime position and could easily make connections to other research areas in the chosen networks. The top 10 research areas with the most frequency in these fields is listed in Table 4. With a frequency of 327, the Cardiac and Cardiovascular Systems was the most influential research area. Peripheral Vascular Disease (frequency: 105) and Medicine, General and Internal (frequency: 91) were the second and third utmost dominant research areas, respectively.

#### Document Citation Analysis

**Figure 12:**
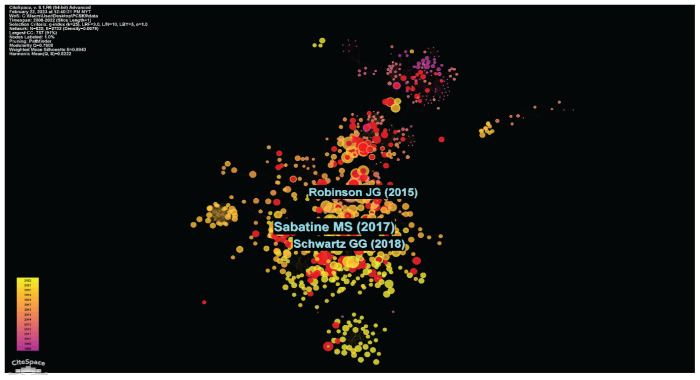
A document co-citation analysis

PCSK9 inhibitors study can discover key or core literature using document co-citation analysis. The complete image of the network of document co-citations is shown in Figure 12. The density of the full network map for this network of document co-citations, which had 825 nodes and 2,702 linkages, was 0.0079. These citation rings show the history of each paper’s citations. The colour of the citation tree rings indicates the referenced time. The thickness in the corresponding time partition is inversely correlated with the number of sources. Sabatine et al. (marked as Sabatine MS, 2017 in Figure 12) from the Brigham and Women’s Hospital and Harvard Medical School, Boston and Schwartz et al. (marked as Schwartz GG, 2018) from the University of Colorado School of Medicine, Aurora, both from the United States were among the utmost significant authors for the document co-citation analysis (i.e. co-cited documents).

#### Document Cluster Analysis

**Figure 13:**
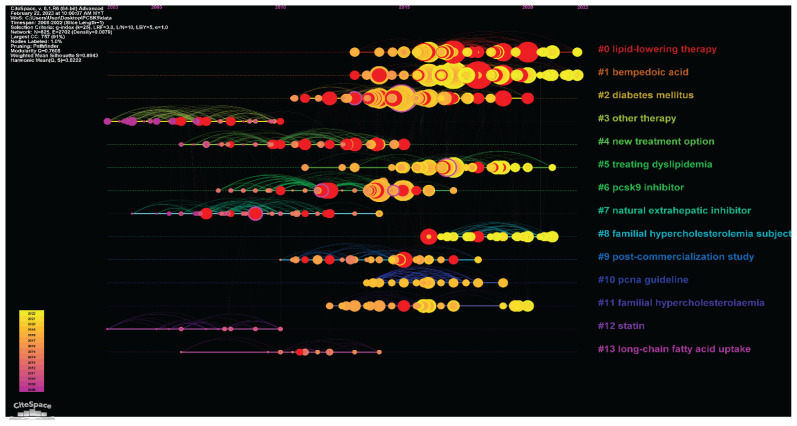
Summary of identified document cluster lifetimes in PCSK9 inhibitors research from 2008 to 2022 generated from the CiteSpace

The complex co-citation network interaction between numerous analysis items is broken down into manageable groups using multivariate statistical analysis techniques, such as cluster analysis. Figure 13 presents the findings from the literature clustering in the form of a timeline fisheye illustration. Intuitively, the cool- to warm-colour change in the citation tree rings represents the ongoing expansion of scientific knowledge. The hottest area of inquiry at the moment is yellow citation tree rings. The 76 primary clusters that comprise the complete network are labelled with index terms from their citations and summarised with the symbol ‘#’ on the right side of Figure 13. It should be emphasised that the clusters are labelled using title phrases and the LLR weighting algorithm. LLR is an algorithm to compute and decide each label’s form, which indicates each cluster’s fundamental notion with professional terms (26). These primary clusters that were isolated and marked with a ‘#’ represent the research boundaries of the discipline’s growth. The 14 significant clusters, each representing a separate research topic, are listed in Table 5. The cluster’s size depends on how many publications are included in it. Cluster #0 (lipid-lowering therapy) has the most 108 publications, followed by cluster #1 (bempedoic acid) has 95 publications and cluster #2 (diabetes mellitus) has 93 publications, respectively. Cluster silhouette scores varied from 0.831 to 0.997, showing a high resemblance between publications within each cluster (silhouette score goes from −1 to 1, with a number greater than 0 indicating homogeneity).

#### Burst Analysis

Trends among studies and keywords are presented, and the most prominent or landmark articles and terms are identified using a burst analysis.

### Document Burst

Table 6 lists the top 10 publications with the most intense citation bursts, with the duration of each burst indicated in the rightmost columns. A burst is the appearance of a keyword in a publication during a specified period. The blue line represents the timeframe (from 2008 to 2022), while the red line represents the burst phase. Four publications, including the top 3, are in the most recent burst. The article ‘Alirocumab and Cardiovascular Outcomes after Acute Coronary Syndrome’ has the robust burst, with a burst strength of 43.66. The study was published in *The New England Journal of Medicine* in 2018, followed by ‘2019 ESC/EAS Guidelines for the management of dyslipidaemias: lipid modification to reduce cardiovascular risk’ (strength = 30.14; burst began in 2020 and ended in 2022), which was published in 2020 in the *European Heart Journal*.

### Keyword Burst

**Figure 14:**
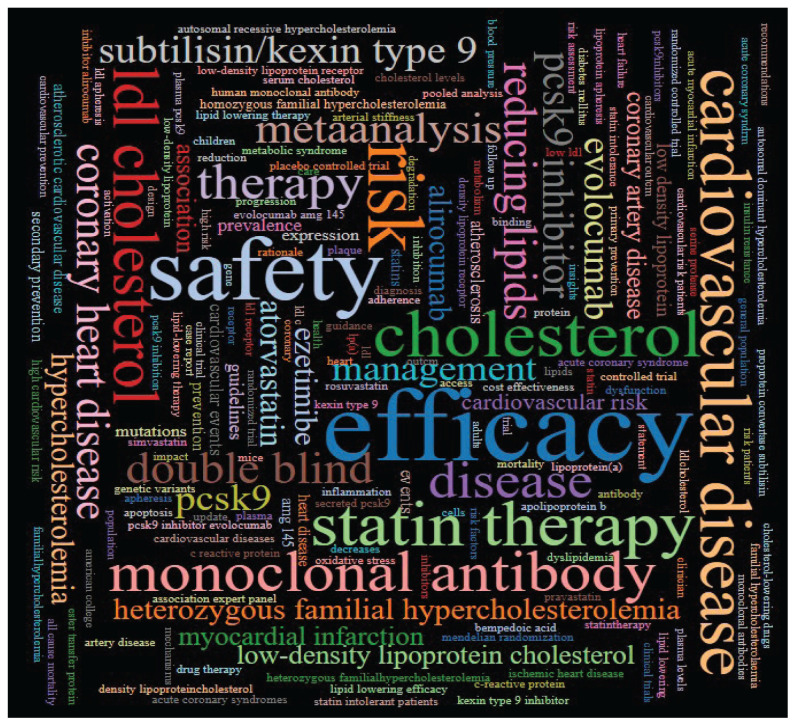
Keyword distribution, created by Bjorn’s Word Cloud by Microsoft Excel 2022 computer worksheet software

The keywords are the enhancement of the main content of the thesis, which can imitate the author’s academic opinions and viewpoints fully. The rapid finding of the keywords refers to the words that are the most recurrently used or used in a shorter period and the distinct attention acknowledged by the scholars at a particular time. Conferring to the word occurrence change of the merged words, the frontiers and trends in this research can be judged. Table 7 lists 10 keywords with the most robust bursts. The last column of the timeline means the entire year of the research (2008–2022), and the red line characterises the period of the keyword outbreak. In terms of bursts strength, the top-ranked is ‘monoclonal antibody’ with bursts of 11.56, followed by ‘reducing lipids’ with bursts of 9.07, occupying the third position is ‘subtilisin/kexin type 9’ (9.0), followed by ‘apolipoprotein b’ (6.77), ‘heterozygous familial hypercholesterolemia’ (6.56), ‘evolocumab AMG 145’ (6.51), ‘autosomal dominant hypercholesterolemia’ (6.32) which bursts strength is all above 6. In Citespace, the strength of a citation burst is calculated based on the occurrence of citations over time. The citation burst analysis in Citespace aims to identify periods of intense citation activity for a specific publication. Briefly, Citespace applies a burst detection algorithm to identify periods of intense citation activity. The algorithm looks for clusters of citations that occur within a relatively short time span compared to the neighbouring citations. These clusters are considered bursts. Once the bursts are detected, Citespace calculates the strength of each burst to quantify its intensity. The strength is calculated based on the number of citations within the burst and the distribution of citations over time within the burst. The strength of a citation burst provides an indication of the impact and influence of a publication during specific time intervals. Publications with higher burst strengths are considered to have experienced more significant citation activity during those periods. Table 8 shows the most often occurring keywords. Figure 14 shows the distribution of the keywords. The word ‘efficacy’ appears 195 times in the article’s title, abstract and keywords, making it the most commonly used term. The word ‘safety’ comes in as number 2 with 169 occurrences, followed by ‘PCSK9 inhibitors’ at number three with 167 events.

## Discussion

Our research aimed to provide a scientometric analysis of the PCSK9 inhibitors domain. This section is devoted to a discussion of our findings. In order to determine the general publication trends in terms of output, a expressive analysis was carried out on the number of articles, published journals, authors, universities/institutes and nations/regions where authors were connected at the time the papers were issued. Over a 5-year period, the number of articles produced has risen steadily, averaging 137 annually. Every year, the number of publications has progressively climbed until reaching 50 in 2016. The country with the most papers published in the field was the United States, followed by England. With 102 total publications, Harvard University in the ‘United States of America’ had the greatest publication rate among the universities. The top 10 organisations and nations varied by region, with advanced countries and prestigious organisations predominating in the elite edition. Asia is only represented by Japan and the People’s Republic of China is among the top 10. We highly urge that more international scientific research interactions and collaboration be performed in the future, despite the possibility that this conclusion results from wealthy countries and prestigious institutions having access to more resources for conducting scientific analysis. A co-citation analysis of the author, journal, nation, institutions, research fields and articles was carried out to identify the key knowledge brokers in these fields. The most significant contributor was Marc Steven Sabatine, an American professor of medicine at Harvard Medical School focusing on cardiovascular medicine. He discusses the monoclonal antibody evolocumab to support its effectiveness and safety in lowering cardiovascular disorders. In predetermined yet exploratory research, Sabatine et al. (27) found that using evolocumab in addition to standard therapy resulted in considerably lower LDL-C levels and a lower incidence of cardiovascular events over a year of treatment (27). According to Sabatine et al. (28), adding evolocumab to statin therapy significantly decreased the risk of cardiovascular events, with a 15% decrease in the risk of the primary composite end point of cardiovascular death, myocardial infarction, stroke, hospitalisation for unstable angina or coronary revascularisation and a 20% decrease in the risk of the more clinically severe key secondary endpoint of cardiovascular death, myocardial infarction or stroke. The proper application of pharmacologic treatments for cardiovascular prevention is the area of expertise of Jennifer G. Robinson, the Emirates Professor of Epidemiology at the University of Iowa in Iowa City. She discusses monoclonal antibodies, primarily alirocumab, to support their effectiveness and safety in lowering the incidence of cardiovascular illnesses. According to Robinson et al. (29) the PCSK9 inhibitor alirocumab fell LDL-C levels in high-risk individuals by an extra 62% points when added to statin therapy at the highest tolerable dose, with or without other lipid-lowering treatment. Professor of Medicine, Gregory G. Schwartz of the University of Colorado School of Medicine is intrigued by clinical trials examining novel lipid and metabolic therapies to enhance outcomes following a heart attack. Patients with a history of the acute coronary syndrome and higher atherogenic lipoproteins despite high-intensity or maximum-tolerated-dose statin therapy showed a reduced risk of severe adverse cardiovascular events when treated with alirocumab compared to placebo, as reported by Schwartz et al. (30). One of the 10 notable authors recently published the ESC/EAS 2019 Lipid Guidelines is François Mach from Switzerland. At the Faculty of Medicine at the University of Geneva in Switzerland, he serves as a full professor and director of the cardiology basic research laboratory. He is a co-chair of the ESC 2021 Prevention Guidelines and one of the three co-chairs of the ESC/EAS 2019 Lipid Guidelines. The two best researchers know each other well and have worked together in the same research group. Some of the 10 most influential authors work a lot in this field (Table 9), and in the last few years, much new research has been done in this area. This shows how important the current approach is and how much more cross-disciplinary work needs to be done in this area.

To connect sub-disciplines, experts in these fields must work with people interested in the same things. Journal content analysis revealed that the fields of Medicine, Clinical Research and Medical Research were the most common. In this field, you can find articles published in high-quality periodicals. *The New England Journal of Medicine*, *Circulation* and the *Journal of the American College of Cardiology* are the top 3 most influential journals in this field. *The New England Journal of Medicine* (NEJM) disseminates the most up-to-date studies and data at the nexus of biological science and clinical practice. This data is presented in clear, clinically applicable formats that guide healthcare decisions and enhance patient outcomes. It has been regularly published for more than 200 years and is acknowledged as the world’s top medical journal and website. Being a publication of the American Heart Association, *Circulation* is a prestigious medical journal. Observational studies, clinical trials, epidemiology, health services, outcomes studies, and developments in basic and translational research are only a few articles published in *Circulation*. The *JACC Family of Journals*, including JACC and nine subspecialty publications, is one of the most read and influential cardiovascular journal series in the world. Each journal in the *JACC Family of Journals* covers a different aspect of cardiovascular medicine, ensuring that researchers, clinicians and experts are always up-to-date on the most cutting-edge research that could impact clinical practice. It was clear from the journal’s emphasis on interdisciplinary work that the framework for the multidisciplinary study had already been laid and that more writers writing in this area were needed. The United States stands above all other countries in this field. The United States’ frequency score is almost three times higher than the scores of the runner-up and third-place countries combined (England and Italy). This shows that influential pieces cited American articles most frequently, whereas foreign publications were quoted almost exclusively by locals. This could cause an ‘Echo Chamber’ effect when only findings from the United States and research undertaken in the country are read, while papers from other countries are ignored. On the other hand, the institution co-citation analysis shows institutions from other nations without deviating from the country results. Among the top 10 institutes, six are from the United States, two are from France, and each is from England and Canada. It is very interesting that not only the academic/medical institutes involved in this field of research but also the pharmaceutical companies play a vital role in discovering PCSK9 inhibitors. This highlights the diversity of research affiliations, with numerous institutions emphasising these subjects. A few researchers in the United States may have dominated this field based on the results of the country and institution. For their research to be well-recognised, other universities outside the nation must speak up more and diversify. Results from outside the United States must be thoroughly shared within this study domain for it to be more advantageous, given that the suppression of PCSK9 serves as the foundation in atherosclerosis treatment. Cardiac and Cardiovascular Systems; Peripheral Vascular Disease; and Medicine, General and Internal, are the top 3 fields of study related to this topic. There is around a three-fold increase in frequency scores for research into cardiac and cardiovascular systems compared to the second and third most popular categories. Some fields of study have a higher centrality than others, indicating that they are strategically placed within the selected networks and may be easily linked to many others. Because the scientifically unique idea is likely to be located in one area, while other regions are followed by articles judged significant by other study areas, this condition may aid the domain’s advancement.

The most often mentioned article, ‘Evolocumab and clinical outcomes in patients with cardiovascular disease,’ referenced 384 times. With a frequency of 203, ‘Alirocumab and cardiovascular outcomes after acute coronary syndrome’ was the second most popular article. The frequency of the third article, ‘efficacy and safety of alirocumab in reducing lipids and cardiovascular events,’ was 176. These pieces emphasise using a monoclonal antibody to lower LDL-C and prevent cardiovascular events. LDL-C levels were reduced by 59% when Evolocumab was used and by 62% when Alirocumab was used. As mentioned earlier, 14 co-citation clusters are just a small subset of the 76 that were discovered via document cluster analysis. Publications were clustered as having a co-citation relationship since other publications cited them. Our research revealed that ‘lipid-lowering therapy,’ ‘bempedoic acid,’ ‘diabetes mellitus,’ and ‘other therapy’ were the most studied and investigated areas. The most important current study cluster is bempedoic acid, one of the clusters. A brand-new, first-in-class oral small chemical called bempedoic acid prevents the creation of cholesterol by controlling the activity of ATP citrate lyase, an enzyme located in the cytosol before 3-hydroxy-3-methylglutarylcoenzyme A reductase. It has so far been tested on patients with and without statin ‘intolerance’ and diabetic patients. Bempedoic acid lowers LDL-C levels by 30% when used alone and roughly 50% when combined with ezetimibe. Phase III trials for bempedoic acid are underway, and some have already been completed. New research topics emerge, and previous burst articles are eventually superseded, as shown by the document burst analysis. The top 10 highest burst scores are from publications published after 2012, which is not too dissimilar from the finding from document co-citation. Since the co-citation score and the burst score are so similar, it’s clear that we need to widen our study scope as new studies arise; this, in turn, calls for either greater resources or a new strategy to ensure that a great deal more high-quality, unique research is published in this field.

## Conclusion

Although using just documents from the WOSCC databases could introduce publication bias, WOSCC is regarded as having higher publication standards due to its database’s focus on the sciences and social sciences, as well as its larger databases with a broader scope than other datasets (38, 39). Comparisons with other databases and WOSCC could be used to create a map of these study topics in the future. There is also the possibility of bias in the dataset due to the fact that we used CiteSpace software instead of manually gathering the data and thus may have accidentally included some irrelevant subjects. On the other hand, thanks to CiteSpace, we can reproduce our findings, and it is challenging to find a middle ground between very restrictive criteria and missing important studies. Future research with a high accuracy target should utilise more stringent keyword searches to lower the probability of irrelevant studies. Finally, this study’s co-citation analyses only included the names of the primary (first) authors. While there was no such limitation on citing publications, WOSCC databases of referenced articles did not retain the identities of other contributing authors. There may be more collaboration than we found if other author identities were available in these databases for the co-citation analysis to produce different results. Notwithstanding these caveats, we believe our work is the most comprehensive review of PCSK9 inhibitors to date. We provide undeniable evidence for the importance of increased global cooperation. We have compiled a body of work in this area from a number of different fields and highlighted important research gaps and potential new avenues of study. Our findings show that there is still a sizable hole that needs to be filled by combined efforts and additional analysis. For instance, the database used contains only one database of WOSCC, reducing the total number of publications that may be evaluated. Further, the study may be lacking because it was conducted in a single language (English) and journals were chosen without grey literature being included.

## Figures and Tables

**Table 1:** Top 10 contributions (i.e. affiliation, journal and author) of PCSK9 inhibitors research worldwide, based on the WOSCC

(A) Affiliation		(B) Journal		(C) Author		

Affiliation name	Counts	Journal name	Counts	Author name	Affiliation name	Counts
Harvard University	102	*Journal of Clinical Lipidology*	61	Sabatine MS	Harvard University	33
Brigham and Women’s Hospital	84	*Atherosclerosis*	36	Giugliano RP	Harvard University	31
Amgen	78	*Journal of the American College of Cardiology*	23	Mach F	University of Geneva	26
Sanofi Aventis	75	*Journal of The American Heart Association*	23	Jukema JW	Leiden University Medical Center (LUMC)	25
Harvard Medical School	73	*European Heart Journal*	19	Pordy R	South Australian Health & Medical Research Institute (SAHMRI)	25
Regeneron	68	*Jama Cardiology*	18	Bhatt DL	Icahn School of Medicine at Mount Sinai	23
Imperial College London	65	*Circulation*	17	Cariou B	Nantes Universite	22
Sanofi France	51	*PLoS ONE*	16	Ray KK	Imperial College London	21
Institut National De La Sante Et De La Recherche Medicale Inserm	46	*European Journal of Preventive Cardiology*	15	Wasserman SM	Brigham & Women’s Hospital	21
Udice French Research Universities	45	*American Journal of Cardiology*	13	Bittner VA	University of Alabama System	19

**Table 2:** Top 10 cited journal based on frequency in PCSK9 inhibitors research

Cited journal	Centrality	Frequency	Year
*New Engl J Med*	0	717	2008
*Circulation*	0.02	618	2008
*J Am Coll Cardiol*	0.01	613	2009
*Eur Heart J*	0.01	562	2011
*Lancet*	0.01	526	2010
*Atherosclerosis*	0.01	523	2008
*JAMA-J Am Med Assoc*	0.01	476	2010
*J Clin Lipidol*	0	387	2009
*Am J Cardiol*	0.05	302	2009
*Arterioscl Throm Vas*	0.02	260	2008

**Table 3:** Top 10 institutions based on the frequency

Institution	Centrality	Frequency	Year
Harvard University	0.1	87	2014
Amgen	0.06	65	2014
Sanofi-Aventis	0.09	57	2014
Brigham and Women’s Hospital	0.05	54	2013
Regeneron	0	41	2014
Imperial College London	0.06	38	2015
Sanofi France	0.02	33	2014
Harvard Medical School	0.04	31	2013
University of Toronto	0.03	29	2017
University of California System	0.05	27	2016

**Table 4:** Top 10 study area co-citation score

Web of Sciences group	Centrality	Frequency	Year
Cardiac and Cardiovascular Systems	0.19	327	2011
Peripheral Vascular Disease	0.15	105	2011
Medicine, General and Internal	0.07	91	2012
Endocrinology and Metabolism	0.01	56	2009
Biochemistry and Molecular Biology	0.20	47	2008
Medicine, Research and Experimental	0.29	44	2015
Health Care Sciences and Services	0.04	26	2016
Multidisciplinary Sciences	0.00	25	2012
Nutrition and Dietetics	0.02	19	2008
Health Policy and Services	0.01	17	2016

**Table 5:** Fourteen significant clusters emerged from document co-citation analysis

Cluster ID	Size	Silhouette	Label (LLR)	Average year
0	108	0.837	lipid-lowering	2017
1	95	0.85	bempedoic acid	2018
2	93	0.851	diabetes mellitus	2015
3	67	0.973	other therapy	2006
4	63	0.831	new treatment option	2011
5	57	0.864	treating dyslipidemia	2017
6	51	0.909	PCSK9 inhibitor	2012
7	46	0.952	natural extrahepatic inhibitor	2008
8	43	0.946	familial hypercholesterolemia subject	2019
9	39	0.955	post-commercialisation study	2014
10	36	0.997	pcna guideline	2015
11	34	0.922	familial hypercholesterolaemia	2015
12	14	0.982	statin	2007
13	11	0.994	long-chain fatty acid uptake	2011

**Table 6:** Top 10 publications with the strongest citation bursts

Title	Journal	Year	Strength	Begin	End	2008–2022
Alirocumab and cardiovascular outcomes after acute coronary syndrome	*The New England Journal of Medicine*	2018	**43.66**	2020	2022	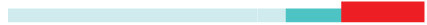
2019 ESC/EAS guidelines for the management of dyslipidaemias: lipid modification to reduce cardiovascular risk	*European Heart Journal*	2020	**30.14**	2020	2022	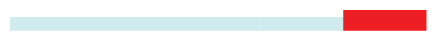
2019 ESC/EAS guidelines for the management of dyslipidaemias: lipid modification to reduce cardiovascular risk	*Atherosclerosis*	2019	**24.99**	2020	2022	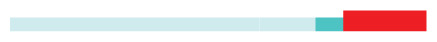
Effect of a monoclonal antibody to PCSK9 on LDL-C	*The New England Journal of Medicine*	2012	**16.06**	2012	2017	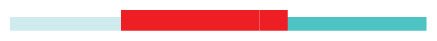
Safety and efficacy of a monoclonal antibody to proprotein convertase subtilisin/kexin type 9 serine protease, SAR236553/REGN727, in patients with primary hypercholesterolemia receiving ongoing stable atorvastatin therapy	*Journal of the American College of Cardiology*	2012	**13.75**	2012	2017	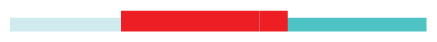
2018AHA/ACC/AACVPR/AAPA/ABC/ACPM/ADA/AGS/APhA/ASPC/NLA/pcna guideline on the management of blood cholesterol: executive summary: a report of the American College of Cardiology/American Heart Association Task Force on Clinical Practice Guidelines	*Journal of the American College of Cardiology*	2019	**13.31**	2020	2022	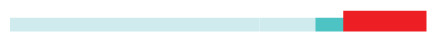
Efficacy and safety of evolocumab in reducing lipids and cardiovascular events	*The New England Journal of Medicine*	2015	**13.22**	2016	2017	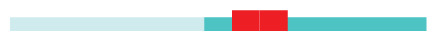
LDL-C-lowering effects of AMG 145, a monoclonal antibody to proprotein convertase subtilisin/kexin type 9 serine protease in patients with heterozygous familial hypercholesterolemia	*Circulation*	2012	**12.96**	2013	2017	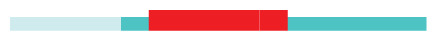
Effects of proprotein convertase subtilisin/kexin type 9 antibodies in adults with hypercholesterolemia	*Annals of Internal Medicine*	2015	**12.92**	2016	2017	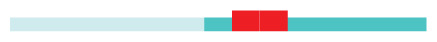
Efficacy, safety, and tolerability of a monoclonal antibody to proprotein convertase subtilisin/kexin type 9 as monotherapy in patients with hypercholesterolaemia (MENDEL): a randomized, double-blind, placebo-controlled, phase 2 study	*The Lancet*	2012	**12.57**	2013	2017	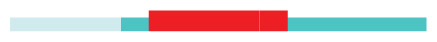

**Table 7:** Top 10 keywords with the strongest citation bursts

Keywords	Year	Strength	Begin	End	2008–2022
Monoclonal antibody	2012	**11.56**	2012	2016	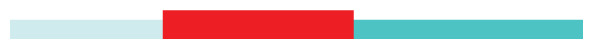
Reducing lipids	2016	**9.07**	2016	2017	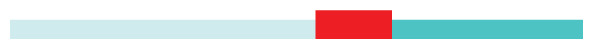
Subtilisin/kexin type 9	2014	**9**	2014	2016	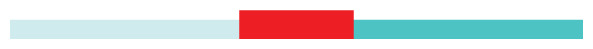
Apolipoprotein b	2009	**6.77**	2009	2017	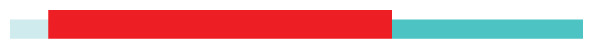
Heterozygous familial hypercholesterolemia	2013	**6.56**	2017	2018	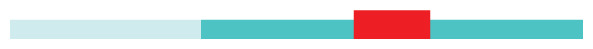
Evolocumab AMG 145	2015	**6.51**	2015	2017	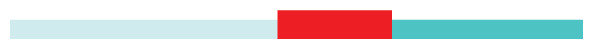
Autosomal dominant hypercholesterolemia	2008	**6.32**	2008	2016	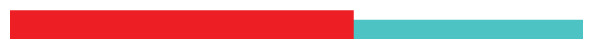
Double-blind	2012	**5.42**	2012	2018	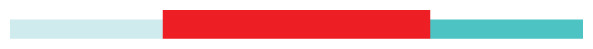
Randomised trial	2015	**5.36**	2015	2018	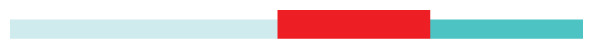
Insights	2020	**5.35**	2020	2022	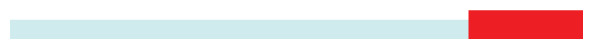

**Table 8:** Top 10 keywords with the highest frequency

Keyword	Frequency
Efficacy	195
Safety	169
PCSK9 inhibitors	167
Risk	137
Cardiovascular disease	128
Cholesterol	127
Familial hypercholesterolemia	123
Statin therapy	121
Density lipoprotein cholesterol	112
LDL-C	112

**Table 9:** Top 10 cited authors based on frequency in PCSK9 inhibitors research

Cited authors	Centrality	Frequency	Year
Sabatine et al. (27)	0.01	483	2015
Robinson et al. (29)	0.03	298	2015
Schwartz et al. (30)	0.02	292	2013
Cannon and Kumar (31)	0.06	266	2009
Baigent et al. (32)	0.05	212	2011
Grundy et al. (33)	0.01	196	2013
Raal et al. (34)	0.03	182	2013
Ridker et al. (35)	0.05	167	2012
Giugliano et al. (36)	0.04	166	2013
Mach et al. (37)	0.00	152	2020
